# The ciliary proteins Meckelin and Jouberin are required for retinoic acid-dependent neural differentiation of mouse embryonic stem cells

**DOI:** 10.1186/2046-2530-1-S1-P77

**Published:** 2012-11-16

**Authors:** S Romani, B Illi, R De Mori, JG Gleeson, EM Valente

**Affiliations:** 1 Istituto Casa Sollievo della Sofferenza- Mendel Laboratory, Italy; 2 Consiglio Nazionale delle Ricerche (CNR), Italy; 3 Howard Hughes Medical Institute, University of California, USA; 4 University of Messina, Department of Medical and Surgical Pediatric, Italy

## 

The dysfunction of the primary cilium, a complex, evolutionarily conserved, organelle playing an important role in sensing and transducing cell signals, is the unifying pathogenetic mechanism of a growing number of diseases collectively termed “ciliopathies”, typically characterized by multiorgan involvement. Developmental defects of the central nervous system (CNS) characterize a subset of ciliopathies showing clinical and genetic overlap, such as Joubert syndrome (JS) and Meckel Syndrome (MS). Although several knock-out mice lacking a variety of ciliary proteins have shown the importance of primary cilia in the development of the brain and CNS-derived structures, developmental in vitro studies, extremely useful to unravel the role of primary cilia along the course of neural differentiation, are still missing. Mouse embryonic stem cells (mESCs) have been recently proven to mimic brain development, giving the unique opportunity to dissect the CNS differentiation process along its sequential steps. In the present study we show that mESCs express the ciliary proteins Meckelin and Jouberin in a developmentally-regulated manner, and that these proteins co-localize with acetylated tubulinlabeled cilia located at the outer embryonic layer. Further, mESCs differentiating along the neuronal lineage activate the cilia-dependent sonic hedgehog signaling machinery, which seems to be impaired in Meckelin knock-down cells but results unaffected in Jouberin-deficient mESCs. However, both seems to lose the ability to acquire a neuronal phenotype. Altogether these findings suggest a pivotal role of Meckelin and Jouberin during embryonic neural specification and indicate mESCs as a suitable tool to investigate the developmental impact of ciliary proteins dysfunction.

